# A CD19/Fc fusion protein for detection of anti-CD19 chimeric antigen receptors

**DOI:** 10.1186/1479-5876-11-23

**Published:** 2013-01-29

**Authors:** Satiro N De Oliveira, Jiexin Wang, Christine Ryan, Sherie L Morrison, Donald B Kohn, Roger P Hollis

**Affiliations:** 1 Department of Pediatrics, University of California, Los Angeles (UCLA), 10833 Le Conte Avenue, A2-410 MDCC, MC 175217, Los Angeles, CA 90095-1752, USA; 2 Department of Microbiology, Immunology and Molecular Genetics (MIMG), University of California, Los Angeles (UCLA), 610 Charles E. Young Dr. East, Los Angeles, CA 90095-1489, USA

**Keywords:** CD19, Chimeric antigen receptor, Fusion protein, Flow cytometry

## Abstract

**Background:**

Chimeric Antigen Receptors (CARs) consist of the antigen-recognition portion of a monoclonal antibody fused to an intracellular signaling domain capable of activating T-cells. CARs displayed on the surface of transduced cells perform non-MHC-restricted antigen recognition and activating intracellular signaling pathways for induction of target cytolysis, cytokine secretion and proliferation. Clinical trials are in progress assessing the use of mature T-lymphocytes transduced with CARs targeting CD19 antigen to treat B-lineage malignancies. CD19 is an attractive target for immunotherapy because of its consistent and specific expression in most of the stages of maturation and malignancies of B-lymphocyte origin, but not on hematopoietic stem cells. Antibodies against the extracellular domain of the CAR molecule (anti-Fab, Fc or idiotype) have been used for detection of CAR expression in research and clinical samples by flow cytometry, but may need development for each construct and present significant background in samples from xenograft models.

**Methods:**

A specific reagent for the detection of anti-CD19 CAR expression was developed, a fusion protein consisting of human CD19 extracellular domains and the Fc region of human IgG1 (CD19sIg). Genes encoding CD19sIg fusion proteins were constructed by fusing either exons 1 to 3 (CD19sIg1-3) or exons 1 to 4 (CD19sIg1-4) of the human CD19 cDNA to a human IgG_1_Fc fragment. These fusion proteins are intended to work in similar fashion as the MHC Tetramers used for identification of antigen-specific T-cells, and may also have other applications in studies of activation of anti-CD19 CAR bearing cells. The CD19sIg proteins were produced from 293 T cells by stable lentiviral vector transduction and purification from culture medium.

**Results:**

ELISA assays using several different monoclonal antibodies to CD19 demonstrated dose-related specific binding by the fusion molecule CD19sIg1-4, but no binding by CD19sIg1-3. Conjugation of the CD19sIg1-4 fusion protein to Alexa Fluor 488 allowed specific and sensitive staining of anti-CD19 CAR-bearing cells for flow cytometry assays, detecting as low as 0.5% of CAR-modified primary cells with minimal background staining.

**Conclusions:**

This fusion molecule is a sensitive reagent for detection of anti-CD19 CAR derived from any monoclonal antibody present in CAR-modified T-cells.

## Background

Chimeric Antigen Receptors (CARs) have been used over the last twenty years to redirect specificity of T-cells for use in immunotherapy research approaches. Among multiple targeted antigens, CD19 has been increasingly studied for its expression in most of the B lineage hematological malignancies, with significant responses in animal models using adoptive transfer of T-cells armed with CD19-specific CAR. The same approach is currently being evaluated in clinical trials with initial success reported [1].

Detection of CAR-bearing cells has usually been performed by flow cytometry, with the use of antibodies against the extracellular structure of the molecule, such as the hinge (using an anti-IgG Fc antibody or F(ab’)_2_ fragment) or the antigen-binding domains (as in the case of the use of an anti-idiotypic antibody). More recently, protein L isolated from Peptostreptococcus was proposed for detection of CAR expression by flow cytometry [2]. Using the antigen specificity of CAR as the determinant of a more specific reagent for the detection of the CD19-specific CAR, we developed a CD19/Fc molecule that can be labeled for its use primarily as a reagent in flow cytometry studies. Our approach consisted of fusing the extracellular domains of the human CD19 protein [3-5] to the human immunoglobulin Fc domain. Fusion to the Fc domain has been used to allow secretion of peptide sequences, with enhanced solubility and stability, and a fusion protein of murine extracellular CD19 and Fc domain has been previously described [6].

We describe the studies for the development and evaluation of this fusion protein. The properties of this reagent make possible sensitive detection by flow cytometry of cells modified with CD19-specific CAR.

## Material and methods

### Construction of CD19-IgG_1_Fc expressing plasmids

The CD19-IgG_1_Fc fusion proteins, CD19sIg1-3 and CD19sIg1-4, were constructed by fusing either exons 1 to 3 (E13) or exons 1 to 4 (E14) of the human CD19 cDNA (Origene, Rockville, MD) to a human IgG_1_Fc (Fc) fragment [7] by PCR-based cloning. Exons 1 through 3 of hCD19 were amplified using primers UNF (5’-CTGGCTAGCGTTTAAACGGG-3’) and X3R (5’-CTGGCTGAGGCTCTGGTTC-3’). Exons 1 through 4 of hCD19 of hCD19 were amplified using primers UNF and X4R (5’- TGGCCGAGCAGTGATCTC-3’). The IgG_1_Fc region was amplified using a 5’phosphorylated primer FCF (5’- TCTGCAGAGCCCAAATCTTG-3’) and the reverse primer ERFC (5’- GTCCAGTGTGGTGGAATTCG -3’). The hCD19 PCR products were digested with EcoR1 and the IgG_1_Fc product was digested with EcoR1. The digested products were ligated into the Fc fragment-containing expression plasmid (pCMV-Fc), previously digested with *Nhe*I and *Eco*R1 to create either pCMV-CD19sIg1-3 or pCMV-CD19sIg1-4. The CD19sIg1-3 and CD19sIg1-4 fragments were then removed from the pCMV-CD19sIg1-3 and pCMV- CD19sIg1-4 expression vectors by restriction enzyme digestion with *Eco*RI and *Xho*I. The lentiviral plasmid pCCLc-EFS-hADA-WPRE (from Adrian Thrasher, University College London, London, UK) contains the short human EF1a promoter [8] and the ADA (adenosine deaminase) transgene. ADA was removed by *Bam*H1 digestion and the backbone religated to make pCCLc-EFS-X-WPRE. The CD19sIg1-3 and CD19sIg1-4 fragments were then cloned into the *Eco*RI/*Xho*I site of the pCCLc-EFS-X-WPRE plasmid to make pCCLc-EFS-CD19sIg1-3-WPRE and pCCLc-EFS-CD19sIg1-4-WPRE.

### Cell culture

293 T cells (ATCC CRL-1268) were cultured in D10: Dulbecco’s modified Eagle’s Medium (DMEM, Mediatech, Herndon, VA) containing 10% fetal bovine serum (Omega Scientific, Tarzana, CA), 50 U/ml penicillin, 50 μg/ml streptomycin, and 2 mM L-glutamine (Gemini Bioproducts, Woodland, CA). Jurkat cells (ATCC TIB-152) and Raji cells (ATCC CCL-86) were cultured in R10: RPMI medium (Irvine Scientific, Santa Ana, CA) containing 10% fetal bovine serum, 50 U/ml penicillin, 50 μg/ml streptomycin, and 2 mM L-glutamine.

### Primary human cells

Human peripheral blood mononuclear cells (PBMC) were obtained from anonymous donor blood samples (through the UCLA CFAR Virology Core Laboratory) and isolated using gradient centrifugation on Ficoll-Hypaque. T-lymphocytes were activated with Dynabeads T-activator CD3/CD28 (Invitrogen, Carlsbad, CA) in RPMI medium containing 10% fetal bovine serum (Omega Scientific, Tarzana, CA), 50 U/ml penicillin, 50 μg/ml streptomycin, and 2 mM L-glutamine (Gemini Bioproducts, West Sacrament, CA) (R10 medium) in an incubator at 37°C and 5% CO_2_. After 72 hours of stimulation, the cells were washed to remove the beads and immediately used for lentiviral transduction, performed without prestimulation or supplemental cytokines in R10 medium, at a vector concentration of 5 × 10^7^ TU/ml. T-lymphocyte cultures were maintained in R10 medium with recombinant human IL- 2 (10 ng/ml) for a minimum of seven days before performing assays. All cultures were tested for T-cell enrichment, and experiments were performed only with populations consisting of at least 85% of CD3-positive cells.

### Primary murine cells

Mouse care and experiments followed protocols approved by the Animal Research Committee at UCLA. For the development of xenografted NSG mice, pups from the strain NOD/SCID/γc^Null^ (NSG) were engrafted with human cells through injection of 2 × 10^5^ CD34(+) human stem/progenitor cells (HSPC) after sub-lethal irradiation (150 rads). Bone marrow from engrafted mice (humanized NSG marrow) and control mice (NSG marrow) was harvested at 14 weeks of age for use in flow cytometry studies.

### Lentiviral vector production

The HIV-1 based lentiviral vectors, CCLc-EFS-CD19sIg1-3-WPRE and CCLc-EFS-CD19sIg1-4-WPRE were produced by triple-plasmid transfection of 5 μg of pCCLc-EFS-CD19sIg1-3-WPRE or pCCLc-EFS-CD19sIg1-4-WPRE plasmid, 5 μg of gag/pol expressing plasmid (pCMVΔR8.91) [9], and 1 μg of the envelope expression plasmid pMD.G (VSV) [10]. The 293 T cells were plated on poly-L-lysine-coated 10-cm plates at 5 × 10^6^ cells per plate in DMEM with 10% FBS (D10) and transfection was performed 24 hours later using the standard TransIT-293 protocol (Mirus Bio, Madison, WI.). Transfected cells were then subjected to induction with 10 mM sodium butyrate (Sigma-Aldrich, St. Louis, MO.) and 20 mM HEPES in D10. After 8–12 hr, the cells were rinsed once with PBS and then fresh D10 with 20 mM HEPES was added. Vector containing supernatant was harvested 48 hours later.

### Concentration, purification and quantification of the fusion protein

For transient expression of CD19-IgG_1_Fc fusion proteins; 1×10^5^ 293 T cells were transfected using the standard manufacturer’s TransIT-293 protocol with 1 μg of pCMV-CD19-IgG_1_Fc plasmid or pCCL-CD19-IgG_1_Fc-WPRE plasmid in 6-well tissue culture plates containing 1 ml of D10 medium. The cells were kept in transfection medium for 24 hours and then cultured in 3 ml of Pro293a™-CDM serum-free medium (LONZA, Basel, Switzerland) containing 2 mM L-glutamine, and then harvested. Stably transduced 293 T cells were seeded in triple-layer flasks (NUNC, Rochester, NY) with 150 ml of normal D10 medium. The medium was changed to 150 ml of Pro293a™-CDM serum-free medium containing 2 mM L-glutamine, when the cells reached confluence. After 2 days, culture medium was harvested and concentrated using Centricon Plus-70 (30 kDa cut-off) centrifugal filter devices (Millipore, Billerica, MA.). Cell lysates were made using NP40 cell lysis buffer (Invitrogen, Carlsbad, CA.). Protease inhibitor cocktail, EDTA free (Thermo Scientific, Waltham, MA) was added to protein samples (supernatant or lysates) right after harvest to prevent degradation. Harvested protein samples were purified using Dynabeads Protein A Immunoprecipitation Kit (Invitrogen, Carlsbad, CA.) according to the manufacturer’s instructions. Elution of the CD19-IgG_1_Fc fusion was performed under denaturing or non-denaturing (native) conditions according to the manufacturer’s instructions. Purified protein samples were quantified by using the Bicinchoninic acid (BCA) assay (Thermo Scientific, Waltham, MA.), using bovine gamma globulin (BGG) for the standard curve.

### SDS PAGE

Non-denaturing protein electrophoresis was performed running 1 μg of each purified protein sample; reducing conditions were performed mixing each purified sample with 1 μl of Sample Reducing Agent (Invitrogen, Carlsbad, CA.) and heating at 70°C for 10 min before electrophoresis on NuPAGE 4-12% Bis-Tris Mini Gels 1.0 mm (Invitrogen, Carlsbad, CA.). The bands were visualized by SimplyBlue™ SafeStain (Invitrogen, Carlsbad, CA.) staining, and the gel was dried using DryEase Mini-Gel Drying System (Invitrogen, Carlsbad, CA.). All procedures were performed according to the manufacturer’s instructions.

### Conjugation of the fusion protein

Purified fusion protein (15–20 μg) was conjugated by using the Alexa Fluor 488 APEX™ Antibody Labeling Kit (Invitrogen, Carlsbad, CA.), according to the manufacturer’s instructions.

### Enzyme-linked immunosorbent assay (ELISA)

Flat-bottomed 96-well plates with a MaxiSorp surface (NUNC, Rochester, NY.) were coated with 100 μL of 10 μg/ml capture antibodies for 1 hour at room temperature. The plates were washed five times with 200 μl phosphate buffered saline with 0.05% Tween-20 (PBST) and then blocked in 300 μl of 5% bovine serum albumin (BSA) (Sigma, St. Louis, MO.) for 1 hour at room temperature. After five washes with PBST, the plates were incubated for 2 h at room temperature with 200 μl of the CD19sIg1-3 or CD19sIg1-4 fusion protein diluted in PBS. Following five washes with PBST, the plates were incubated for 1 hour at room temperature with 100 μl of alkaline phosphatase (AP)-conjugated detection antibodies. Finally, the plates were washed five times with PBST before being developed for 30 minutes at room temperature with 100 μl of a p-Nitrophenyl-phosphate (pNPP) substrate (Sigma, St. Louis, MO.) and stopped by equal volume of 0.75 M NaOH. The stopped reactions were assayed by spectrophotometry at 405 nm. Capture antibodies used included goat anti-human IgG Fc polyclonal antibody (Millipore, Billerica, MA.); Mouse anti-human CD19 monoclonal antibodies including: FMC63 (Millipore, Billerica, MA.), 2E2B6B10 (Abcam, Cambridge, MA.), and F-3 (Santa Cruz Biotechnology, Santa Cruz, CA.) and HIB19 (BD Biosciences, San Jose, CA); Mouse anti-human CD20 B9E9 (Santa Cruz Biotechnology, Santa Cruz, CA); and Mouse anti-human PSMA (Prostate-Specific Membrane Antigen) YPSMA-1 (Abcam, Cambridge, MA.). The detection antibody used was goat anti-human IgG Fc polyclonal antibody AP (Millipore, Billerica, MA) (1:5000).

### Flow cytometry

Transduced Jurkat cells and primary T-cells expressing the anti-CD19 CAR were detected in mixed samples by incubating 2×10^5^ cells with 450 ng of the labeled fusion protein at 4°C for 30 minutes in the dark, after being blocked by human serum from AB plasma (Sigma-Aldrich) for 10 minutes. After being washed two times with PBS, cells were analyzed on a LSR II (BD Biosciences, San Jose, CA.) machine running the FACSDiva™ software (BD Biosciences, San Jose, CA.). Similar staining protocol was used for the commercially available CD19-Fc fusion protein, rhCD19-Fc (ACROBiosystems, Bethesda, MD.). Staining with biotinylated Protein L (GenScript, Piscataway, NJ.) was performed immediately after the blocking procedure following manufacturer instructions, and secondary labeling was performed with FITC-Streptavidin (Sigma). For all tests, FITC-conjugated F(ab’)_2_ fragment goat anti-human IgG1 Fc_γ_ (Jackson ImmunoResearch Laboratories, West Grove, PA.) (55.5 ng per 10^5^ cells) was used as the positive control for the detection of anti-CD19 CAR positive cells, after incubation of the cell samples in 5% FBS for 10 minutes.

CD19-specific blocking experiments were performed incubating labeled CD19sIg1-4 fusion protein with FMC63 for 1 hour at 4°C before being added to the cells for staining, at concentrations described in the results section. The staining conditions after pretreatment were kept 450 ng of Alexa Fluor 488-labeled CD19sIg1-4 for 2×10^5^ cells.

## Results

### Construction of CD19-IgG1Fc expressing plasmids and lentiviral vector production

**Figure 1:**
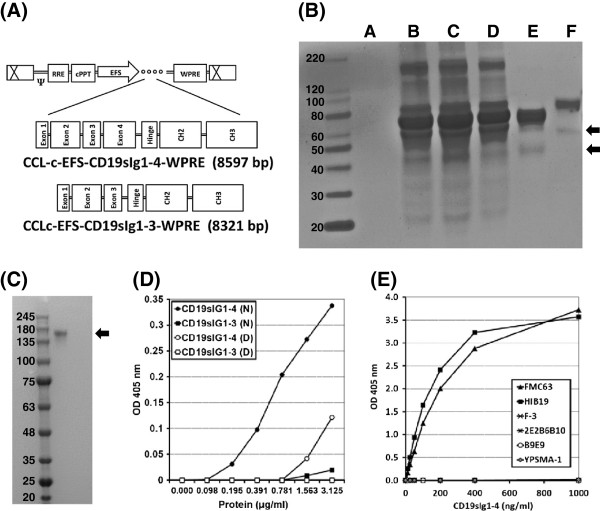
**Construction and characterization of the CD19-IgG**_**1**_**Fc fusion proteins. A**. Diagrams of the lentiviral vector provirus constructs used to transduce the CD19sIg fusion genes. **B**. Reduced protein electrophoresis by SDS PAGE of fusion protein products after concentration and purification using Protein A Dynabeads. Centricon concentrated supernatant samples from fresh Pro293a™-CDM **(A)**, 293 T cells **(B)**, 293 T cells expressing CD19sIg1-3 **(C)**, and 293 T cells expressing CD19sIg1-4 **(D)**. Lanes E and F are the DYNAL purified extracts for CD19sIg1-3 and CD19sIg1-4 (respectively). Arrows indicate the predicted size of the monomer, 47 kDa for CD19sIg1-3 and 57 kDa for CD19sIg1-4. All samples were reduced prior to loading. **C**. Native protein electrophoresis of CD19sIg1-4 after concentration and purification using Protein A Dynabeads; arrow points to 171 kDa band (expected size for trimers of the fusion protein). **D**. Results of a comparative ELISA using FMC63 monoclonal capture antibody, of purified fusion proteins, in native (N) or denatured **(D)** forms. **E**. Results of ELISA using antibodies targeting human CD19 molecule (FMC63, HIB19, F-3 and 2E2B6B10), human CD20 (B9E9) and PSMA (YPSMA-1).

The extracellular domain for human CD19 protein contains two immunoglobulin-like domains and is coded by the exons 1 through 4 [3-5]. The fusion proteins containing the CD19 extracellular domain were designed in two versions, fusing exons 1–3 (CD19sIg1-3) and exons 1–4 (CD19sIg1-4), to human IgG1 fragment (Figure 1A); this strategy was chosen as previously published data suggested that the addition of exon 4 decreased protein secretion (deFougerolles et al., 2001). The fusion fragments were cloned into the pCCL lentiviral backbone driven by the EFS promoter (Figure 1A).

### Establishment of producer cell line

Lentiviral vectors were packaged and then used for stable transduction of 293 T cells. Clones were selected by highest level of production of fusion proteins assayed by ELISA. The best-transduced clones produced approximately double the amount of fusion protein (35.49 μg/ml of supernatant of CD19sIg1-3 and 16.91 μg/ml of CD19sIg1-4) yielded by transiently transfected 293 T cells (17.99 and 10.78 μg/ml of supernatant). Cell lysates had fusion protein yields of ten to twenty times more compared to the supernatant harvest, but this extracted protein was denatured and contained higher amounts of extraneous proteins.

### Concentration & purification of the fusion proteins

Forty-eight hours before supernatant harvest, the fusion protein-expressing clones were cultivated in serum-free medium, in order to avoid interference of the serum proteins in the purification process. Centricon filters with 30 kDa cut-off were used to concentrate the cell supernatant, and Protein A Dynabeads were used for purification of the fusion proteins, as Protein A binds to the Fc portion of CD19sIg1-3 and CD19sIg1-4.

### Product characterization

From calculations based on the amino acid sequences alone, the fusion proteins CD19sIg1-3 and CD19sIg1-4 should have molecular masses respectively of 47 kDa and 57 kDa. Figure 1B shows a reduced SDS-PAGE electrophoresis gel with the fusion proteins isolated from producer cell supernatants, confirming expected findings. Depending on the pH of the eluate from the Protein A Dynabeads purification process, the fusion proteins were isolated in the presence or absence of denaturing conditions. Denatured samples were kept at pH 2.8 and native samples had their pH re-adjusted to 7.5 immediately following non-denaturing elution. Figure 1C shows a non-denaturing protein electrophoresis of CD19sIg1-4 after Protein A purification. These different conditions did not influence the fusion protein mobility on SDS-PAGE (unlike sample reduction, data not shown), but affected the functional assays, as shown on Figure 1D. However, in addition to the expected monomer-sized band observed on Figure 1B, the majority of the protein is larger and in which likely represents glycosylated or dimeric forms, slowing the mobility on the gel.

### ELISA assays

ELISA assays were used to assess the presence of necessary CD19 epitopes on the CD19sIg1-3 and CD19sIg1-4 fusion proteins, using as a capture antibody the murine monoclonal FMC63 (Figure 1D), the same monoclonal antibody used for development of most of the CD19-specific CAR constructs currently used in clinical trials [11]. CD19sIg1-3 presented minimal binding, and only CD19sIg1-4 showed FMC63 specific binding, in both denatured and native forms. To further investigate the CD19 epitopes on CD19sIg1-4, different antibodies targeting the human CD19 molecule were analyzed by ELISA as capture antibodies (HIB19, F-3 and 2E2B6B10). As a control, antibodies targeting human CD20 and prostate-specific membrane antigen (PSMA), two previously published targets of CAR, were also evaluated against CD19sIg1-4 (Figure 1E). As expected, the anti-CD20 and anti-PSMA antibodies did not demonstrate any binding of the CD19sIg1-4 fusion protein, and FMC63 and HIB19 bound to CD19sIg1-4 in a dose-dependent fashion. F-3 and 2E2B6B10 did not bind to the fusion protein. F-3 is an antibody directed to the C-terminus, absent in the fusion protein, and 2E2B6B10 is an antibody developed against full-length recombinant human CD19, recommended for immunohistochemistry, ELISA, Western Blot, but not flow cytometry, therefore most likely also targeting the C-terminus. These results present evidence that the binding sites of the FMC63 and HIB19 antibodies are the extracellular domains (N-terminus) of the human CD19 protein present, at least in part, in CD19sIg1-4.

### Flow cytometry staining using fusion proteins

**Figure 2:**
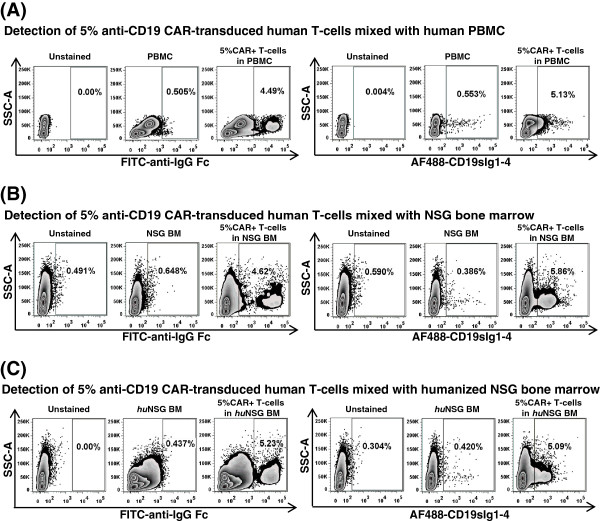
**Evaluation of CD19sIg1-4 fusion protein on primary human cell populations.** Flow cytometry plots using FITC-conjugated anti-IgG Fc F(ab’)_2_ fragment (FITC-anti-IgG Fc, left panels) or Alexa Fluor 488-labeled CD19sIg1-4 (AF488-CD19sIg1-4, right panels) for detection of 5% anti-CD19 CAR-transduced human primary T-cells mixed with **(A)** human peripheral blood mononuclear cells (PBMC), **(B)** NSG bone marrow (NSG BM), and **(C)** humanized NSG bone marrow (*hu*NSG BM).

The isolated fusion protein CD19sIg1-4 was conjugated to Alexa Fluor 488 and used to detect Jurkat cells transduced to stably express CD19-specific CAR by flow cytometry (data not shown). To investigate the ability of the reagent to specifically detect CD19-specific CAR-expressing primary human cells and to evaluate the presence of undesirable non-specific background staining, mixtures of cells were prepared for flow cytometry acquisitions. These included CAR-positive primary human T-cells mixed with normal donor human PBMC (Figure 2A), murine NSG marrow (Figure 2B) and “humanized” NSG marrow from NSG mice engrafted with human CD34(+) cells (Figure 2C). Acquisition gating strategies were purposely broad in order to evaluate the heterogeneous cell populations, and not just lymphocytes. In each experiment, samples stained with the labeled fusion protein presented distinct homogeneous staining of CAR-modified cells, with less background staining than the FITC-conjugated anti-IgG Fc F(ab’)_2_ fragment. In Figure 2C, non-specific binding of FITC-anti-IgG Fc F(ab’)_2_ fragment to “humanized” NSG marrow cells required gate adjustment in order to allow proper detection of CAR-modified cells. Labeled CD19sIg1-4 was a reliable reagent for the detection of CD19-specific CAR-modified cells, with minimal binding to human PBMC, and virtually no binding to murine or humanized murine bone marrow cells, unlike the FITC-conjugated anti-IgG Fc F(ab’)_2_ fragment.

**Figure 3:**
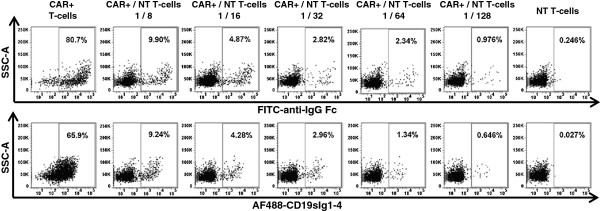
**Evaluation of CD19sIg1-4 fusion protein for detection of anti-CD19 CAR-modified primary human T-cells.** Flow cytometry plots demonstrating the sensitivity of detection of anti-CD19 CAR-transduced human primary T-cells mixed in increasing numbers of non-transduced (NT) T-cells using FITC-conjugated anti-IgG Fc F(ab’)_2_ fragment (FITC-anti-IgG Fc, upper panels) or Alexa Fluor 488-labeled CD19sIg1-4 (AF488-CD19sIg1-4, lower panels).

To evaluate the sensitivity of detection using the fusion protein CD19sIg1-4, T-cells were activated from PBMC and transduced with a lentiviral vector carrying a CD19-specific CAR derived from the FMC63 monoclonal antibody [12]. CAR-transduced populations of T-cells with 75% of anti-CD19 CAR expression were mixed with increasing numbers of non-transduced (NT) T-cells, creating a range of percentages of CAR-modified T-cells between 0.5 and 75%, and those cell mixes were stained with either FITC-conjugated anti-IgG Fc F(ab’)_2_ fragment (Figure 3, upper panels) or Alexa Fluor 488-labeled CD19sIg1-4 (Figure 3, lower panels). The efficiency of detection of CAR-expressing cells with CD19sIg1-4 was similar to the detection using FITC-conjugated F(ab’)_2_ fragment goat anti-human IgG1 Fc_γ_. Detection with labeled CD19sIg1-4 was reliable even with as low as 0.5% of modified cells, a level of detection below what is needed for efficacy in immunotherapy models. Detection of CD19-specific CAR-transduced cells revealed MFI for FITC-conjugated F(ab’)_2_ anti-IgG1 Fc_γ_ of 15,065 and MFI for Alexa Fluor 488-labeled CD19sIg1-4 of 2,848, at similar detection rates. The decreased MFI with the CD19sIg1-4 protein may be due to the heterogeneous labeling of the fusion protein with Alexa Fluor 488. Another possible explanation is that the binding avidity of CD19 for the scFv of anti-CD19 CAR is lower than the binding of the FITC-conjugated F(ab’)_2_ anti-IgG1 Fc_γ_.

**Figure 4:**
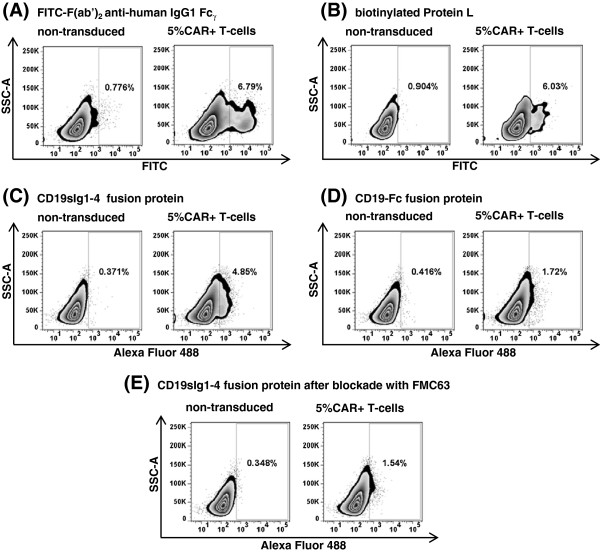
**Comparison of CD19sIg1-4 fusion protein to similar commercially available reagents.** Flow cytometry plots of staining of primary human T-cells, non-transduced and 5% CAR-transduced, using FITC-conjugated F(ab’)_2_ fragment goat anti-human IgG1 Fc_γ_**(A)**, biotinylated Protein L **(B)**, Alexa Fluor 488-labeled CD19sIg1-4 **(C)** and Alexa Fluor 488-labeled rhCD19-Fc fusion protein **(D)**. **E**. Results of staining of the same cell population after pre-incubation of Alexa Fluor 488-labeled CD19sIg1-4 with anti-CD19 monoclonal antibody FMC63.

In order to evaluate the performance of CD19sIg1-4 against other similar commercially available reagents, anti-CD19 CAR-positive primary human T-cells at expected frequency of 5% mixed with non-transduced T-cells were stained with Alexa Fluor 488-labeled CD19sIg1-4, Alexa Fluor 488-labeled rhCD19-Fc fusion protein (ACROBiosystems), biotinylated Protein L (GenScript) and FITC-conjugated F(ab’)_2_ fragment goat anti-human IgG1 Fc_γ_ (Figure 4). Staining of the cell population by FITC-conjugated F(ab’)_2_ anti-IgG1 Fc_γ_ was once again the brightest, with mean detection of 6.8% CAR-modified cells. Biotinylated Protein L presented mean detection of 6.4% of cells, while CD19sIg1-4 had mean detection of 4.8%, compared to 1.6% by the CD19-Fc fusion protein from ACROBiosystems. Similarly to previous results, significant staining background was observed after staining with FITC-conjugated F(ab’)_2_ anti-IgG1 Fc_γ_ and Protein L (demonstrated on non-transduced T-cells panels on Figure 4A and 4B). Both CD19sIg1-4 and CD19-Fc fusion proteins labeled with Alexa Fluor 488 presented similar MFI (Figure 4C and 4D).

To provide further evidence of specific binding, CD19 blocking experiments were performed using anti-human CD19 monoclonal antibody FMC63. Alexa Fluor 488-labeled CD19sIg1-4 (40 ng/μl) was mixed with FMC63 (500 ng/μl) at 12.5 : 1 ratio and incubated for 1 hour at 4°C before being added to staining tubes. The flow cytometry results demonstrated a 70% detection decrease (Figure 4E, from 4.8% to 1.5%), confirming that the binding of CD19sIg1-4 to CAR-modified cells is specifically mediated by anti-CD19 and CD19 interaction.

## Discussion

The fusion protein CD19sIg1-4 presented higher binding to the anti-CD19 monoclonal antibody FMC63 than CD19sIg1-3 (Figure 1D), and for this reason it was chosen for progression into the next series of experiments. The exons 2 and 4 of the human CD19 gene code for two C2-type immunoglobulin-like domains, separated by a small intervening domain coded in exon 3; we hypothesize that the absence of exon 4 in the CD19sIg1-3 construct may have affected the final protein structure and epitope recognition by FMC63. CD19sIg1-4 also avidly bound to other anti-CD19 monoclonal antibodies, and did not bind to monoclonal antibodies specific to other antigens, further demonstrating evidence that the human CD19 molecule was present in its structure. The binding of CD19sIg1-4 to Protein A also demonstrates that Fc domains (that bind to Protein A) are constitutively present in the fusion protein.

The detection specificity of labeled CD19sIg1-4 was evident even in complex cell mixtures (Figures 2A-C). Previously published studies on flow cytometric detection of CAR expression have routinely used enriched T-cell populations, minimizing staining background. Comparative staining using same population of anti-CD19 CAR-transduced T-cells with CD19sIg1-4 against other commercially available reagents, biotinylated Protein L, CD19-Fc fusion protein, and FITC-conjugated F(ab’)2 fragment goat anti-human IgG1 Fc, demonstrated consistent and sensitive detection of CAR-modified T-cells (Figures 3 and 4) with less non-specific staining. The commercially available CD19-Fc fusion protein (ACROBiosystems) differs from CD19sIg1-4 as it does not contain all of exon 1 and contains part of exon 5; we hypothesize the detection performance of that reagent may have been impaired by distinct epitope pattern, by distinct polymerization, by the labeling procedure with Alexa Fluor 488, or a combination of all. Commonly used alternative approaches, such as anti-human IgG1 Fc or biotinylated Protein L, present significant background and limited discrimination. Protein L presents the need of secondary staining with labeled streptavidin, with additional protocol steps and potential cell loss. The findings described here provide evidence of superior performance of the labeled fusion protein CD19sIg1-4 in detecting CD19-specific CAR-positive cells, even in complex cell populations, as found in common experimental situations for evaluation of CAR-mediated cytotoxicity in tumor immunotherapy models and clinical trials.

## Conclusions

A fusion protein containing the extracellular domain of the human CD19 molecule fused to IgG1Fc was developed and stably produced and secreted by a modified cell line. CD19sIg1-4 was recognized by two α-CD19 antibodies and bound specifically by CD19-specific CAR-expressing cells. Detection of CD19-specific CAR-expressing cells by the CD19sIg1-4 fusion protein was very sensitive (detecting populations as low as 0.5%) and specific, superior to currently commercially available CD19-Fc fusion protein, and comparable to standard detection with FITC-conjugated F(ab’)_2_ fragment goat anti-human IgG1 Fc_γ_ and biotinylated Protein L, with reduced background staining when used in samples containing primary hematopoietic cells.

## Abbreviations

CAR: Chimeric antigen receptor; CD: Cluster of differentiation; Fc: Fragment crystallizable region of an antibody; Fab: Fragment antigen-binding region of an antibody; HSPC: Hematopoietic stem/progenitor cells; MFI: Mean fluorescence Intensity; NSG: Mouse strain strain NOD/SCID/γc^Null^; PBMC: Peripheral blood mononuclear cells; PSMA: Prostate-specific membrane antigen.

## Competing interest

The authors declare that they have no competing interests.

## Authors’ contributions

SD carried out the flow cytometry assays, participated in the conception and design and drafted the manuscript. JW carried out the molecular cloning, protein isolation and characterization. CR carried out the cell cultures and immunoassays. SM participated in the conception and design of the protein isolation and characterization. DK participated in the experimental conception and design, data analysis and interpretation and manuscript drafting. RH participated in the experimental conception and design, protein isolation and characterization, and manuscript drafting. All authors read and approved the final manuscript.
